# The Quiet Eye and Motor Expertise: Explaining the “Efficiency Paradox”

**DOI:** 10.3389/fpsyg.2018.00104

**Published:** 2018-02-08

**Authors:** André Klostermann, Ernst-Joachim Hossner

**Affiliations:** Institute of Sport Science, University of Bern, Bern, Switzerland

**Keywords:** gaze behavior, motor learning, quiet eye, task-solution space, inhibition hypothesis

## Abstract

It has been consistently reported that experts show longer quiet eye (QE) durations when compared to near-experts and novices. However, this finding is rather paradoxical as motor expertise is characterized by an economization of motor-control processes rather than by a prolongation in response programming, a suggested explanatory mechanism of the QE phenomenon. Therefore, an inhibition hypothesis was proposed that suggests an inhibition of non-optimal task solutions over movement parametrization, which is particularly necessary in experts due to the great extent and high density of their experienced task-solution space. In the current study, the effect of the task-solution space’ extension was tested by comparing the QE-duration gains in groups that trained a far-aiming task with a small number (low-extent) vs. a large number (high-extent) of task variants. After an extensive training period of more than 750 trials, both groups showed superior performance in post-test and retention test when compared to pretest and longer QE durations in post-test when compared to pretest. However, the QE durations dropped to baseline values at retention. Finally, the expected additional gain in QE duration for the high-extent group was not found and thus, the assumption of long QE durations due to an extended task-solution space was not confirmed. The findings were (by tendency) more in line with the density explanation of the *inhibition hypothesis*. This density argument suits research revealing a high specificity of motor skills in experts thus providing worthwhile options for future research on the paradoxical relation between the QE and motor expertise.

## Introduction

Expertise in sport is characterized by consistently superior performance of an athlete over a long period of time (e.g., Starkes, 1993). Based on the great efforts that have been put toward the study of motor-skill learning over the last decades (for an overview, e.g., Baker and Farrow, 2015), superior visual behavior has been identified as a hallmark of expertise (e.g., Ericsson, 2017). In this regard, experts show more fixations of longer durations on task-relevant areas and, conversely, fewer fixations on task-irrelevant areas. In addition, experts utilize longer saccades and shorter fixation latencies to task-relevant objects (Mann et al., 2007; Gegenfurtner et al., 2011).

The quiet eye (QE) – defined as the final fixation or tracking gaze at a task-relevant location prior to the initiation of the final phase of the movement (Vickers, 2007) – is a phenomenon that exemplifies expertise-related differences in fixation behavior (Vickers, 1996). In a typical QE study, Causer et al. (2010) investigated the visual behavior of elite and sub-elite athletes in trap shooting. They found longer relative QE durations for hits (*M* = 60.7%) than for misses (*M* = 56.5%). Moreover, elite shooters showed longer relative QE durations (*M* = 62.6%) than their less-skilled counterparts (*M* = 54.7%). Likewise, Causer et al. (2017) reported longer QE durations in trials with a low (*M* = 1180 ms) than in trials with a high radial error (*M* = 845 ms) in a golf-putting task. To date, the QE has been studied in more than 25 different motor tasks (Vickers, 2016) and a number of reviews (e.g., Causer et al., 2012; Wilson et al., 2015) as well as meta-analyses (Mann et al., 2007; Lebeau et al., 2016; Rienhoff et al., 2016) suggest the significance of this phenomenon.

Despite the robustness of the empirically identified phenomenon and some progress over the recent years (for an overview, e.g., Gonzalez et al., 2015), the mechanisms underlying the QE effect are still not well-understood. This particularly concerns the paradoxical finding of increasing QE durations with increasing motor expertise that was labeled the “efficiency paradox” by Mann et al. (2016). On the one hand, this paradox is based on the observation that motor expertise is generally characterized by an economisation of behavior and an “automatization” of underlying control processes (e.g., Fitts and Posner, 1967). Such an efficiency increase is, for example, reported by Maslovat et al. (2011) who showed decreased reaction times – indicating decreased processing demands – in retention tests after learning a one-handed aiming task (for an overview, see McMorris and Graydon, 2000). On the other hand, with respect to the QE, Williams et al. (2002) explain their finding of increased QE durations in billiards as a function of task difficulty, with increasing demands for the fine-tuning of the movement. However, if expertise is characterized by an economisation of control processes and if, as suggested by Williams et al. (2002), the QE reflects the time needed for information processing over motor control, then a reduction rather than an extension of the QE duration should be expected with growing expertise.

Consequently, Klostermann et al. (2014a) proposed an alternative explanation of the QE phenomenon that is still rooted in the cognitive domain but does not emphasize the amount of information that needs to be processed over the QE interval. Drawing on the selection-for-action mechanism proposed by Neumann (1996; see also Allport, 1987, as well as Cisek and Kalaska, 2010), rather a “shielding mechanism” over the QE period is suggested that inhibits the preparation of non-optimal task solutions such that only the optimal movement variant is executed. To this effect, the QE would simultaneously support the continuous process of action selection from the distributed representations of response options (see also Cisek and Kalaska, 2010, p. 278). On the basis of this functionality, it can be hypothesized that the increasing number of alternative task solutions gathered over years of practice comes with increasing shielding demands that, in turn, lead to the prediction of longer QE durations for experts than for novices or near-experts. Hence, the *inhibition hypothesis* as proposed by Klostermann et al. (2014a) offers a straightforward explanation to the finding of increasing QE durations with increasing motor expertise.

When attempting to empirically test the *inhibition hypothesis*, first off, one must elaborate in which way the assumed shielding process might be hindered by a task-solution space of an experienced expert. In this regard, two variables become relevant. On the one hand, the QE of an expert might be increased due to the *extension* of his/her task-solution space, meaning that task variants far from the “standard” solution had been experienced in such a way that these solutions are combined in one single space. On the other hand, the QE duration might also be prolonged as a function of the *density* of the task-solution space, meaning that a lot of different task variants very close to the “standard” solution had been experienced that thus allow the expert to better fine-tune the movement and perform the task with low variance.

Findings recently reported by Horn et al. (2012) can be interpreted to support the extent explanation of the *inhibition hypothesis*. In their study, participants performing a dart-throwing task with random practice showed longer QE durations than participants with a blocked-practice protocol. Because random practice is suggested to enhance the formation of rules over the entire task-solution space (Magill and Hall, 1990), random practice can be understood as extending the gathered experience over the task-solution sub-spaces. When illustrating this argument with the example of basketball throws from a variety of different positions: if throws from positions A, B, and C are practiced in a blocked fashion, players can be expected to form separate rules for each position which results in separate task-solution sub-spaces for positions A, B, and C. If, however, the positions are randomly varied, players can be expected to conceive the positions as belonging to one and the same task which results in the formation of rules for one single task-solution space. It should be noted that the two players do not differ with respect to their individual space’s density but rather regarding the extent of the abstracted space. When being required to perform a throw from position B, the player with the more extended task-solution space then needs to shield the current movement variant against more alternative solutions than the player with the less extended task-solution space. Consequently, on the basis of the *inhibition hypothesis*, longer QE durations can be expected for random practice than for blocked practice.

However, since Horn et al. (2012) only measured performance effects, it remains unclear whether the reported findings also hold for motor learning. Consequently, the current study sought to extend the findings of Horn et al. (2012) by (1) introducing a retention test. Further, considering the expertise-related context, (2) a significant prolongation of the learning phase seemed advisable. Finally, (3) more differentiated treatments were compared that better meet the specific requirements of the extent explanation. To this end, two groups of participants trained a far-aiming ball-throwing task with the non-dominant hand. Whilst the low-extent group practiced a small number of task variants in a block-wise fashion as in the Horn et al. (2012) study, the high-extent group trained a large number of task variants, which were presented in a structured rather than a random order to further push participants to abstract rules over the entire task-solution space rather than over separate subspaces (see Hossner et al., 2016). The main prediction for the group comparison concerns the QE variable, as we expected longer durations in post-test and retention test for the high-extent group when compared to the low-extent group. In order to guard this prediction from potential contamination by confounding variables, task variants needed to be chosen for the test phases that could be expected to lead to comparable amounts of learning for both groups. Therefore, with regards to motor learning, it was only predicted that both groups improved performance from pretest to post-test and retention test. In cases of performance differences, however, this effect would be needed to be considered as a confounding variable, meaning that a more pronounced QE extension of the high-extent group could alternatively be explained by the higher motor expertise.

## Materials and Methods

### Participants

Nineteen male (age: 22.5 ± 1.4 years) and 11 female (age: 21.0 ± 1.0 years) right-handed sport-science students volunteered in the study and received course credits in return. The participants were assigned to one of two intervention groups on the basis of their pretest throwing performance and gaze behavior. All participants had self-reported normal or corrected-to-normal vision, and all were unaware of the research question. Written informed consent from the participants were obtained in advance. This study was carried out in accordance with the 1964 Declaration of Helsinki. The protocol was approved by the ethics committee of the Faculty of Human Sciences of the University of Bern.

### Apparatus

A 10-camera Vicon-T20 system (200 Hz, VICON Motion Systems Limited, Oxford, United Kingdom) assessed participants’ throwing performance as well as the movements of the throwing arm. For this reason, balls were manufactured from retro-reflective fabric that is detectable by the VICON cameras and a rigid cluster composed of four retro-reflective markers was attached to the throwing arm.

The gaze behavior was assessed with a mobile eye-tracker (220 Hz, EyeSeeCam, EyeSeeTec GmbH, Fürstenfeldbruck, Germany). For power supply and data transfer, the EyeSeeCam was connected via an active FireWire extension (GOF-Repeater 800, Unibrain, San Ramon, CA, United States) to a MacBook Pro (Apple, Cupertino, CA, United States), which was connected to the VICON workstation for the synchronization of EyeSeeCam and VICON data. Three additional VICON markers attached to the EyeSeeCam recorded the three-dimensional (3D) translation and rotation of the participant’s head. Combining the head movements with the vertical and horizontal rotations of the left eye – assessed by the EyeSeeCam via reflection of infrared light from the pupil and the cornea – a 3D gaze vector was calculated in the laboratory frame of reference. The accuracy of the EyeSeeCam system amounts to 0.5° of visual angle with a resolution of 0.01° RMS within 25° of the participant’s field of view (Kredel et al., 2015).

At the beginning of each test session, the EyeSeeCam was calibrated by consecutively fixating five dots. The positions of the dots were calculated based on the current 3D translation and rotation of the participants head and were then accordingly displayed on a life-size screen (height: 2.0 m, width: 3.5 m) with gaps of 8.5° of visual angle between horizontally or vertically neighboring dots. The accuracy of the gaze measurement was verified at the beginning and halfway through each test block of 16 trials. The EyeSeeCam was recalibrated if the point of gaze deviated more than 1° of visual angle from one of the points of the calibration grid.

The target stimuli to be hit were displayed on a life-size screen (height: 2.0 m, width: 3.5 m) with an LCD projector (Epson H271B LCD Projector, Nagano, Japan). Standing with their feet shoulder-width apart, the participants were positioned at a distance of 3.1 m to the screen. On their right side, a box was positioned at hip height that contained numerous retro-reflective balls (50 mm in diameter). At a distance of 2 m behind the participants, two loudspeakers (Microspot Multimedia CP 250, Microspot, Moosseedof, Switzerland) were installed that played audio stimuli for signaling the beginning of the each throwing attempt, thereby forcing participants not to hasten through the data-acquisition phase and rather focus on accuracy in each single trial.

All stimuli were programmed with Mathworks Matlab 2016a (The Mathworks, Inc., Natick, MA, United States) and rendered with Magix Video Pro X3 (Magix Software GmbH, Berlin, Germany). Data analyses were conducted with Mathworks Matlab 2016a and IBM SPSS Statistics 24 (IBM, Armonk, NY, United States).

### Procedure

**FIGURE 1:**
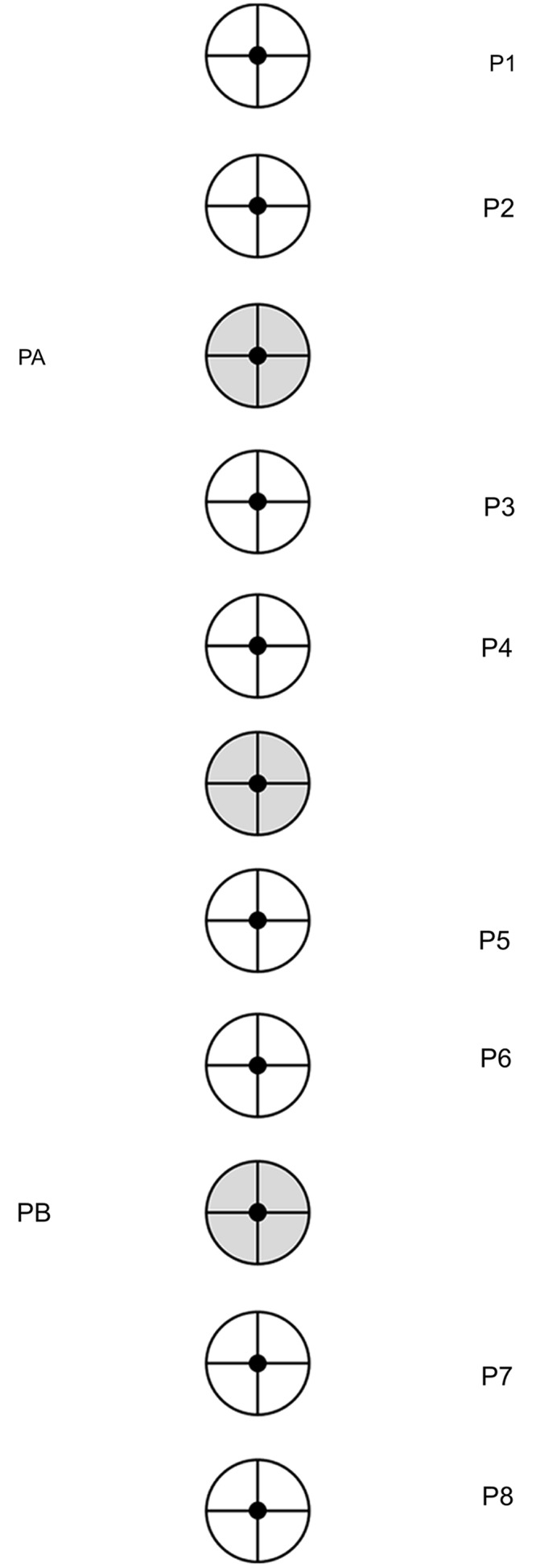
Target positions for the practice phases of the high-extent group (P1–P8) and the low-extent group (PA, PB) as well as for the test phases (highlighted in gray). In the beginning of each trial, a fixation point was presented either to the left or the right for 1000 ms (black dots). Following an audio signal, the fixation point disappeared and the target for the current trial was presented for 6000 ms.

The study was conducted in the institute’s sensorimotor laboratory in which participants had to attend 10 individual sessions of about 45 min each. After having read the instructions, the participants were equipped with the marker cluster and the EyeSeeCam. Following the first calibration, the test or intervention session started. Participants’ task was to always throw a ball as precisely as possible at a target (size: 240 mm in diameter) by performing a pendulum-like underhand throwing technique with the non-dominant (i.e., left) hand. As depicted in **Figure 1**, 11 targets were used that were arranged in a vertical line on the screen at equal distances of 200 mm and at heights ranging from 2200 mm (P1) to 200 mm (P8). In the practice sessions, two targets (PA, PB) were used for the low-extent group and eight targets (P1–P8) for the high-extent group. In the test sessions, both groups had half of the trials aimed at the training targets of the low-extent group (upper target: 1800 mm; lower target: 600 mm) and the other half at a target that had been trained by neither the low-extent nor the high-extent groups (middle target: 1200 ms).

In the first and last session, respectively, the pre- and retention tests were conducted. After a warm-up block of eight trials (2 x upper/lower targets, 4 x middle target; random order), two test blocks of 16 trials each were executed (4 x upper/lower targets, 8 x middle target; quasi-randomized order with each target appearing not more than three times in a row). In each test trial, a fixation cross was presented for 1000 ms at the height of the middle target PC either 900 mm to the left or to the right of the vertical line of the screen (randomized order; see **Figure 1**). Followed by an audio signal, the current target was presented at one of three positions (upper/middle/lower target). The target disappeared after 6000 ms to prevent any time pressure of the participants.

Beginning with the second session, the group-specific interventions commenced, with six blocks of 16 trials per session, resulting in a total of 768 intervention trials per participant. For the high-extent group, the targets P1–P8 were presented in a structured order by moving stepwise through the task-solution space from top to bottom and back again in each block. For the low-extent group, only targets PA and PB were presented in a blocked order by beginning each block with eight trials aimed at PA before changing to PB. The last intervention session was completed with the post-test, which was conducted after a short break following the last intervention trial and was structured as described above for the pre- and retention tests.

Due to the time-consuming intervention phase as well as restricted availabilities, individual schedules needed to be coordinated with each participant. Resulting from these arrangements, the first intervention session was conducted about 6 days after the pretest session (low-extent group: 5.7 ± 0.8 days; high-extent group: 6.1 ± 0.8 days), about 4 days elapsed between each of the eight intervention sessions (both intervention groups: 4.3 ± 0.1 days), and the retention test session followed about 5 days after the post-test (low-extent group: 4.9 ± 0.4 days; high-extent group: 4.7 ± 0.4 days). After the retention test, the participants were thanked and debriefed about the objectives of the study.

### Measures

Trials with technical difficulties in data collection (pretest: 0.4%; post-test: 1.1%; retention test: 2.8%) and trials without a valid QE registration (pretest: 4.9%; post-test: 11.2%; retention test: 6.6%) had to be excluded from further data analyses. In addition, one participant from the low-extent group was not able to complete the intervention due to an injury and thus had to be removed from the analyses.

#### Throwing Performance

Throwing performance was obtained by computing radial-error scores. To this end, for each trial, the position of the ball at the moment of ball impact and the position of the center of the target disk were assessed, with the latter computed by converting the relative position of the target in the video scenes into the screen frame of reference. The moment of impact was detected by the negative peak in the ball’s acceleration curve (cf. Klostermann et al., 2014b). For the pretest, post-test, and retention test, the performance measure aggregated all 32 test trials.

#### Quiet Eye

The QE measure was derived from both the gaze data and the synchronized kinematic data of the throwing movement. To this end, the raw gaze data were first filtered with a Median Bandpass Filter (window size: 10 frames, cut-off frequencies 1 and 10 Hz) and the kinematic data of the throwing arm’s marker cluster were smoothed with a 41 point, 3rd order Savitzky–Golay filter. From the resulting 3D gaze data in the laboratory frame of reference, a screen-intersection point was calculated to provide a gaze location in the screen frame of reference for each time step (i.e., 5 ms). By use of a dispersion-based algorithm (Nyström and Holmqvist, 2010), fixations were identified if the resulting gaze path was stable within an area of 1.2° of visual angle for at least 120 ms. The QE duration was defined as the duration of the final fixation at the target before the initiation of the forward swing which, in turn, was determined as the first instant in time the average position of the arm marker cluster moved forward after having reached the backmost position (cf. Klostermann et al., 2014b). For the pretest, post-test, and retention test, the average QE duration was calculated from the total 32 test trials.

### Statistical Analyses

QE duration and throwing performance were analyzed with mixed-factorial ANOVAs with time of measurement (3) as the within-participant factor and intervention group (2) as the between-participant factor. In cases of sphericity assumption violations, Greenhouse–Geisser corrections were applied. *A posteriori* effect sizes were computed as partial eta squared, $\eta_{p}^{2}$, and Cohen’s *d*-values.

## Results

### Throwing Performance

**FIGURE 2:**
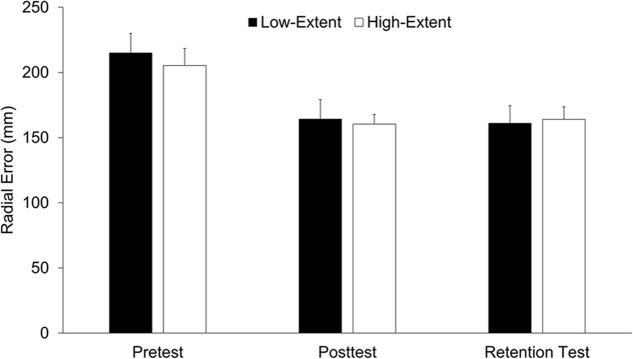
Throwing performance of the two intervention groups (low-extent vs. high-extent) as a function of time of measurement (pretest, post-test, retention test). Error bars indicate standard error.

As illustrated in **Figure 2**, both intervention groups improved throwing performance from pre- to post-test and maintained performance in retention. Consequently, a main effect for time of measurement was revealed, *F*(1.37,36.95) = 29.45, *p* < 0.05, $\eta_{p}^{2}$ = 0.52, with significantly more accurate throws in post-test and retention when compared to pretest (all *p*s < 0.05, all *d*s > 1.2), but no significant differences between post-test and retention test (*p* = 0.93, *d* < 0.01). Further, main and interaction effects failed to reach the pre-determined level of significance (all *p*s > 0.68, all $\eta_{p}^{2}$< 0.01). In particular, the error scores in post-test and retention test did not differ between groups (all *p*s > 0.82, all *d*s < 0.08, all 1-β < 0.08).

### Quiet Eye

**FIGURE 3:**
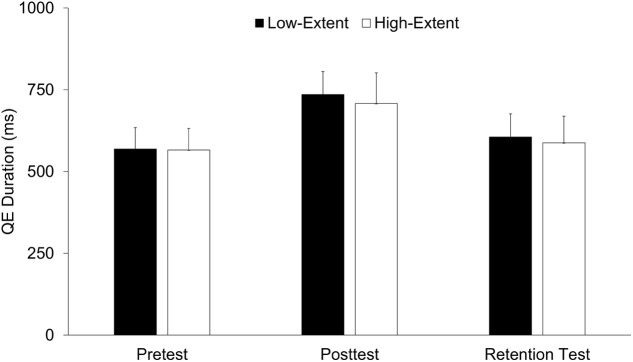
Quiet eye (QE) duration of the two intervention groups (low-extent vs. high-extent) as a function of time of measurement (pretest, post-test, retention test). Error bars indicate standard error.

As shown in **Figure 3**, a main effect for time of measurement was revealed for QE duration, *F*(1.61,43.45) = 5.09, *p* < 0.05, $\eta_{p}^{2}$ = 0.16. Independent of the intervention, participants had longer QE durations in post-test when compared to pretest and retention test (all *p*s < 0.05, all *d*s > 0.58). No significant difference was found between pretest and retention test, *t*(29) = 0.48, *p* = 0.63, *d* = 0.10. Further main and interaction effects were non-significant (all *p*s > 0.87, all $\eta_{p}^{2}$< 0.01).

## Discussion

The classical finding of longer QE durations with increasing motor expertise seems rather paradoxical, especially when considering the suggestion of optimized information processing caused by a QE prolongation. However, the *inhibition hypothesis* offers an explanation for this paradox as it relates the better explored task-solution space of experts to the increased requirement to shield the optimal movement variant against alternative movement parametrisations. The hypothesis that the QE is needed to finalize this shielding process was tested by comparing the QE durations of two groups with different extents of task-solution sub-spaces after extensive practice. More precisely, we expected the participants of a low-extent group, due to their blocked-practice treatment, to abstract rules for separate subspaces whilst the structured-practice treatment of the participants of a high-extent group was expected to result in the abstraction of rules for one single task-solution space. As a consequence of the higher extent of the task-solution space, the requirements regarding the shielding of the current task variant against – a larger number of – alternatives was expected to be higher for the high-extent than for the low-extent group. Hence, we predicted longer QE durations in post-test and retention test for the high-extent group when compared to the low-extent group.

The performance results showed that both groups threw more accurately in post-test and retention test, illustrating a stable motor-learning effect for both groups. The fact that both groups did not differ in performance after learning confirms the successful implementation of fair learning and test conditions. Consequently, the QE findings can be discussed exclusively with regards to the experimental manipulations, which should be highlighted because superior skill acquisition in one or the other groups would have been a strong alternative explanation for respective QE differences.

As predicted, participants showed longer QE durations in post-test when compared to the pretest, however, this gain completely vanished in the retention test. First, this unexpected finding implies that the results reported by Horn et al. (2012) should be interpreted in terms of performance but not as learning effects. Consequently, the longer QE durations revealed for the random-practice group in their study might indeed reflect increased response-programming demands, but cannot be understood as being caused by a behaviorally stable QE extension (see also, e.g., Williams et al., 2002). Second, the instability of the QE effect was surprising, in particular, because earlier training studies with similar retention intervals quite consistently reported QE-learning effects. For example, Vine and Wilson (2010) trained novices in golf putting either with coupled QE-technique instructions or with technical instructions only. Both groups showed stable QE durations in the 2-day-delayed and in the 5-day-delayed retention test. However, unlike the study at hand, the technically-instructed participants also received guidance to maintain head stability after club-ball contact (p. 366). Thus, it might be speculated that long-lasting effects in QE learning depend on respective verbal guidance during training. Consequently, from a practical viewpoint, future research should give further consideration to this relation.

It could be argued that the unexpected lack of temporally stable QE effects might devastate the basic rationale of the present study because the missing “efficiency paradox” at the retention test might not allow to explain the paradox for principle reasons. Indeed, we acknowledge that an intervention-induced QE prolongation in conjunction with a performance improvement would have been highly desirable. However, it also should be noticed that the present study focuses on a hypothetical mechanism underlying a QE prolongation and, as a matter of course, the lack of an empirically found QE prolongation does not rule out that certain aspects of the supposed mechanism are empirically detectable. Hence, we would like to state that it is still worthwhile to discuss group differences in the present study because it might be that the experimentally induced differences in inhibition demands result in measurable differences on the level of the dependent variable (especially in the post-test). However, when comparing the two intervention groups based on this argument, it needs to be recognized that also the core finding of this study was not in line with our prediction because both groups did not differ in QE duration, neither in the post-test nor in the retention test and even after a considerably extensive training phase of more than 750 trials. Of course, this negative result cannot be taken as empirical support of the inhibition hypothesis.

Two explanation can be offered for this lack of group differences. First, it may be argued that 750 practice trials did not suffice to stimulate the formation of different task-solution spaces. The counter-argument against this way of thinking would be that, in other studies, QE effects have been found with far less amount of practice trials (e.g., 320 trials in Vine and Wilson, 2010). Nevertheless, the present study aimed on considerably smaller differences in the group treatments so that the amount-of-practice argument must be acknowledged.

However, a second explanation for the absence of group differences should also be considered. This explanation refers to the fact that the present study focused on question whether the *extension* of the task-solution space – to a lower or a higher degree – affects inhibition demands and, in turn, QE durations. Thus, it might not be the extent factor that best explains the assumed increased inhibition demands, but rather the *density* factor, meaning that it would be less important that experts form elaborate rules that cover the entire (large extent) task-solution space. Inhibition would rather be needed on a more fine-grained level *within* sub-spaces in which the acquired experiences are densely packed such that it is particularly hard to shield the chosen movement variant against immediately neighboring alternatives.

It can be argued that this latter explanation is even – at least by tendency – supported by the data at hand because the low-extent group, for which a denser exploration of the task-solution space around the practiced targets can be expected than for the high-extent group, shows a slightly longer QE duration (∼30 ms on average) in post-test and retention test when compared to the high-extent group. As additionally calculated, this difference particularly surfaces when separately analyzing the trained (upper/lower) and the non-trained (middle) targets, with the effect appearing by tendency more pronouncedly for the trained targets (∼50 ms on average). Thus, it could be speculated that the high amount of practice condensed the task-solution space in the specifically practiced regions. However, as already argued above, the total amount of about 750 practice trials might not have been enough to yield a significant effect in the present study. When comparatively considering, for instance, the 100s of hours NBA players practice free-throws, this experience can definitely be expected to result in a very dense task-solution sub-space due to the massive amount of broadly similar task executions. Consequently, in order to perform this task at the highest level, it may still be hypothesized that a long QE period is needed to shield the finally chosen task solution against very similar but less successful variants.

It should be noted that the density argument developed would also be well in line with research in the motor-performance domain in which a high specificity of motor skills in experts was revealed. For example, Keetch et al. (2005) found that basketball players taking free-throw shots from the original distance performed better than would be predicted by the relationship of the accuracies of set shots attempted at different distances. This especial skill is assumed to represent a very specific, well-learned movement pattern, a “general motor program” (Keetch et al., 2008) which, in the context at hand, implies a dense task-solution sub-space. Regarding the above-sketched relation to the QE duration, this effect would lead to the prediction that QE durations in expert basketball players should increase as a function of task demands (i.e., with increased distance to the basket, cf. Williams et al., 2002). However, referring to the density assumption of the *inhibition hypothesis*, it can also be expected that the QE duration at the immensely practiced free-throw distance should be longer than would be predicted by the relationship among other throwing distances. Such experiments are well-planned for implementation in the near future.

## Author Contributions

All authors listed have made a substantial, direct and intellectual contribution to the work, and approved it for publication.

## Conflict of Interest Statement

The authors declare that the research was conducted in the absence of any commercial or financial relationships that could be construed as a potential conflict of interest.

## References

[B1] Allport A. (1987). “Selection for action: some behavioral and neurophysiological considerations of attention and action,” in *Perspectives on Perception and Action* eds Heuer H. Sanders A. F. (Hillsdale, NJ: Lawrence Erlbaum), 395–419.

[B2] Baker J. Farrow D. (2015). *Routledge Handbook of Sport Expertise.* London: Routledge.

[B3] Causer J. Bennett S. J. Holmes P. S. Janelle C. M. Williams A. M. (2010). Quiet eye duration and gun motion in elite shotgun shooting. *Med. Sci. Sports Exerc.* 42 1599–1608. 10.1249/MSS.0b013e3181d1b059 20139787

[B4] Causer J. Hayes S. J. Hooper J. M. Bennett S. J. (2017). Quiet eye facilitates sensorimotor preprograming and online control of precision aiming in golf putting. *Cogn. Process.* 18 47–54. 10.1007/s10339-016-0783-4 27822605 PMC5306057

[B5] Causer J. Janelle C. M. Vickers J. N. Williams A. M. (2012). “Perceptual expertise: what can be changed?” in *Skill Acquisition in Sport* eds Hodges N. Williams A. M. (London: Routledge) 306–324.

[B6] Cisek P. Kalaska J. F. (2010). Neural mechanisms for interacting with a world full of action choices. *Annu. Rev. Neurosci.* 33 269–298. 10.1146/annurev.neuro.051508.135409 20345247

[B7] Ericsson K. A. (2017). Expertise and individual differences: the search for the structure and acquisition of experts’ superior performance. *Wiley Interdiscip. Rev. Cogn. Sci.* 8:e1382 10.1002/wcs.1382. 27906512

[B8] Fitts P. M. Posner M. I. (1967). *Human Performance.* Oxford: Brooks and Cole.

[B9] Gegenfurtner A. Lehtinen E. Säljö R. (2011). Expertise differences in the comprehension of visualizations: a meta-analysis of eye-tracking research in professional domains. *Educ. Psychol. Rev.* 23 523–552. 10.1007/s10648-011-9174-7

[B10] Gonzalez C. C. Causer J. Miall R. C. Grey M. J. Humphreys G. Williams A. M. (2015). Identifying the causal mechanisms of the quiet eye. *Eur. J. Sport Sci.* 17 74–84. 10.1080/17461391.2015.1075595 26356536

[B11] Horn R. H. Okumura M. S. Alexander M. G. F. Gardin F. A. Silvester C. T. (2012). Quiet eye duration is responsive to variability of practice and to the axis of target changes. *Res. Q. Exerc. Sport* 83 204–211. 10.1080/02701367.2012.10599851 22808706

[B12] Hossner E.-J. Käch B. Enz J. (2016). On the optimal degree of fluctuations in practice for motor learning. *Hum. Mov. Sci.* 47 231–239. 10.1016/j.humov.2015.06.007 26123921

[B13] Keetch K. M. Lee T. D. Schmidt R. A. (2008). Especial skills: specificity embedded within generality. *J. Sport Exerc. Psychol.* 30 723–736. 10.1123/jsep.30.6.723 19164838

[B14] Keetch K. M. Schmidt R. A. Lee T. D. Young D. E. (2005). Especial skills: their emergence with massive amounts of practice. *J. Exp. Psychol. Hum. Percept. Perform.* 31 970–978. 10.1037/0096-1523.31.5.970 16262492

[B15] Klostermann A. Kredel R. Hossner E.-J. (2014a). On the interaction of attentional focus and gaze: the quiet eye inhibits focus-related performance decrements. *J. Sport Exerc. Psychol.* 36 392–400. 10.1123/jsep.2013-0273 25226608

[B16] Klostermann A. Kredel R. Hossner E.-J. (2014b). The quiet eye without a target: the primacy of visual information processing. *J. Exp. Psychol. Hum. Percept. Perform.* 40 2167–2178. 10.1037/a0038222 25314047

[B17] Kredel R. Klostermann A. Hossner E.-J. (2015). “Automated vector-based gaze analysis for perception-action diagnostics,” in *Advances in Visual Perception Research*, ed. Heinen T. (New York, NY: Nova Science Publisher) 45–59.

[B18] Lebeau J. C. Liu S. Sáenz-Moncaleano C. Sanduvete-Chaves S. Chacón-Moscoso S. Becker B. J. (2016). Quiet eye and performance in sport: a meta-analysis. *J. Sport Exerc. Psychol.* 38 441–457. 10.1123/jsep.2015-0123 27633956

[B19] Magill R. A. Hall K. G. (1990). A review of the contextual interference effect in motor skill acquisition. *Hum. Mov. Sci.* 9 241–289. 10.1080/17461391.2014.957727 25252156

[B20] Mann D. T. Y. Williams A. M. Ward P. Janelle C. (2007). Perceptual-cognitive expertise in sport: a meta-analysis. *J. Sport Exerc. Psychol.* 29 457–478. 10.1123/jsep.29.4.457 17968048

[B21] Mann D. T. Y. Wright A. Janelle C. M. (2016). Quiet eye: the efficiency paradox – comment on vickers. *Curr. Issues Sport Sci.* 1 111–114. 10.15203/CISS-2016.111

[B22] Maslovat D. Hodges N. J. Chua R. Franks I. M. (2011). Motor preparation and the effects of practice: evidence from startle. *Behav. Neurosci.* 125 226–240. 10.1037/a0022567 21280935

[B23] McMorris T. Graydon J. (2000). The effect of incremental exercise on cognitive performance. *Int. J. Sport Psychol.* 31 66–81.

[B24] Neumann O. (1996). “Theories of attention,” in *Handbook of Perception and Action* eds Neumann O. Sanders F. (San Diego, CA: Academic Press) 389–446.

[B25] Nyström M. Holmqvist K. (2010). An adaptive algorithm for fixation, saccade, and glissade detection in eyetracking data. *Behav. Res. Methods* 42 188–204. 10.3758/BRM.42.1.188 20160299

[B26] Rienhoff R. Tirp J. Strauß B. Baker J. Schorer J. (2016). The ‘quiet eye’and motor performance: a systematic review based on newell’s constraints-led model. *Sports Med.* 46 589–603. 10.1007/s40279-015-0442-4 26712511

[B27] Starkes J. L. (1993). Motor experts: opening thoughts. *Adv. Psychol.* 102 3–16. 10.1016/S0166-4115(08)61462-4

[B28] Vickers J. N. (1996). Visual control when aiming at a far target. *J. Exp. Psychol. Hum. Percept. Perform.* 22 342–354. 10.1037/0096-1523.22.2.342 8934848

[B29] Vickers J. N. (2007). *Perception, Cognition and Decision Training: The Quiet Eye in Action.* Champaign, IL: Human Kinetics.

[B30] Vickers J. N. (2016). Origins and current issues in quiet eye research. *Curr. Issues Sport Sci.* 1:101 10.15203/CISS-2016.101

[B31] Vine S. J. Wilson M. R. (2010). Quiet eye training: effects on learning and performance under pressure. *J. Appl. Sport Psychol.* 22 361–376. 10.1080/10413200.2010.495106

[B32] Williams A. M. Singer R. N. Frehlich S. G. (2002). Quiet eye duration, expertise, and task complexity in near and far aiming tasks. *J. Mot. Behav.* 34 197–207. 10.1080/00222890209601941 12057892

[B33] Wilson M. Causer J. Vickers J. N. (2015). “Aiming for excellence: the quiet eye as a characteristic of expertise,” in *Routledge Handbook of Expertise* eds Baker J. Farrow D. (New York, NY: Routledge) 22–37.

